# Is early-phase amyloid PET ready for use as neurodegeneration marker?

**DOI:** 10.1177/13872877251366671

**Published:** 2025-08-14

**Authors:** Cecilia Boccalini, Valentina Garibotto

**Affiliations:** 1 Laboratory of Neuroimaging and Innovative Molecular Tracers (NIMTlab), Geneva University Neurocenter and Faculty of Medicine, University of Geneva, Geneva, Switzerland; 2 Division of Nuclear Medicine and Molecular Imaging, Geneva University Hospitals, Geneva, Switzerland; 3 CIBM Center for Biomedical Imaging, Geneva, Switzerland

**Keywords:** Alzheimer's disease, dementia, imaging

## Abstract

Alzheimer's disease is marked by accumulation of amyloid-β (Aβ) and abnormal tau, associated with subsequent neurodegeneration. Validated markers of neurodegeneration include brain glucose metabolism, perfusion and atrophy. Dual-phase amyloid PET imaging allows to assess both Aβ buildup and perfusion changes through a single tracer injection. Increasing evidence supports the comparability of early-phase amyloid PET scans and glucose metabolism changes assessed by ^18^F-FDG PET at group and individual levels, regardless of the amyloid tracers and the disease, emphasizing the added value of dual-phase amyloid PET imaging protocols for clinical and research settings.

Amyloid PET, which assesses brain amyloid deposition, and ^18^F-fluorodeoxyglucose (^18^F-FDG) PET, which assesses glucose metabolism, provide valuable and complementary information for the diagnosis of Alzheimer's disease (AD) and other neurodegenerative diseases.^1^ Extensive research indicates that integrating multiple biomarkers—whether PET-based or not—enhances diagnostic precision.^2^ While a recent set of diagnostic criteria proposes that a positive amyloid status, assessed by amyloid PET or fluid biomarkers, could be necessary and sufficient for AD diagnosis,^3^ ^18^F-FDG PET as measure of neurodegeneration adds important steps in terms of disease staging, clinical outcomes and presence of other pathologies.^4^ ^18^F-FDG PET also allows assessment of the extent and location of hypometabolism specific for neurodegenerative diseases other than AD, helping in differential diagnosis and giving insight about heterogeneity.^2^ The importance of this additional information is well consolidated in the recent intersocietal recommendations for a biomarker-based diagnosis of neurocognitive disorders, which gives a prominent role to ^18^F-FDG PET.^2^

Dual-phase protocol for amyloid PET data acquisition has proven to be able to evaluate brain amyloidosis and neurodegeneration at the same time, adding to the reference late acquisition a short acquisition of the tracer distribution immediately after injection. The optimal early time frame has been usually investigated based on the best within- or inter-subject correlation with ^18^F-FDG PET, achieving the best association with a 5- to 10-min acquisition with some tracer-related differences.^5–7^ In this way, dual-phase protocol provides a perfusion measure very close to metabolism through the early-phase scan because of the high lipophilicity of the tracers^8,9^ and the neurovascular coupling for which cerebral perfusion is strongly related to metabolic consumption in aging and dementia conditions.^10,11^ Using different amyloid tracers, early-phase amyloid PET has demonstrated strong concordance with ^18^F-FDG PET uptake at the group level, supporting its potential as a surrogate marker for neuronal dysfunction in AD and non-AD.^5–7,12–20^ Some differences in underlying biological mechanisms and regional uptakes between the two modalities have been recognized without impacting on the overall good concordance. For instance, ^18^F-FDG-PET has a slow and steady uptake, while blood flow has a high extraction in the first passage, which is however tracer-specific. ^18^F-FDG-PET is associated with active cellular uptake, likely involving glial cells, which may not be observed in perfusion examinations.^21,22^ Regarding regional differences between the two modalities, low cerebellar metabolism was found on ^18^F-FDG-PET and higher medial temporal perfusion and perfusion in the thalamus and the brainstem in early-phase amyloid PET.^23^

The applicability of dual-phase amyloid PET at single-subject level has been first demonstrated using ^18^F-Florbetapir and ^18^F-Flutemetamol tracers, showing a comparable distribution of perfusion to metabolism and a comparable ability in excluding the presence of neurodegeneration.^24^ In line with the strong relationship between hypometabolism and cognitive performance,^4^ perfusion also showed a comparable good correlation with Mini-Mental State Examination scores.^24^ Lojo-Ramírez and colleagues confirmed the applicability of dual-phase amyloid-PET at single-subject level also using ^18^F-Florbetaben.^25^ They further supported the robustness of early-phase ^18^F-Florbetaben PET as a surrogate biomarker for neurodegeneration, independent of amyloid and clinical status. Indeed, the hypoperfusion patterns found by single-subject voxel-wise t-maps identified neurodegenerative patterns similar to those seen in ^18^F-FDG PET, allowing neuroimaging experts to have the same rating for two image modalities and supporting clinical translation.^25^ An important added value of this additional measure of neurodegeneration is the ability to assess, in amyloid positive cases, the presence of neurodegeneration that allows linking the underlying pathology with the presence of clinical signs and symptoms, while in amyloid negative cases the presence or absence of specific hypoperfusion patterns can provide elements supporting alternative degenerative or non-degenerative etiologies.^19^

The sample included in the study by Lojo-Ramirez et al.^25^ evaluated mainly prodromal and dementia patients, leaving open the question about the suitability of early-phase PET for individuals in preclinical phases, such as subjective cognitive decline, when brain metabolic changes can be already present^26^ and ^18^F-FDG PET might be more accurate.^24^ In this framework, the sensitivity of early-phase amyloid PET to predict clinical progression in a specific preclinical population needs to be addressed.

Other open issues still need to be considered. Semi-quantification is strongly recommended in conjunction with visual reading by several clinical consensus, the official recommendations by the Society of Nuclear Medicine and Molecular Imaging (SNMMI) and the European Association of Nuclear Medicine (EANM), to perform high-quality reads of ^18^F-FDG-PET in the clinical setting and freeware and commercial software are available on different methods for ^18^F-FDG-PET. Among semi-quantification methods, voxel-based single-subject analysis has been suggested in common practice guidelines for supporting visual interpretation of brain ^18^F-FDG-PET,^27,28^ and a comparable approach is likewise desirable for perfusion imaging. However, a healthy control (HC) dataset is essential for achieving strong performance in voxel-wise analyses and methods for early-phase images are less developed compared to those for ^18^F-FDG-PET. The use of ^18^F-FDG-PET HC datasets for the analysis of early-phase images has been tested but is suboptimal given the systematic differences in regional uptakes of the tracers. Although artificial intelligence strategies have been tested to correct these systematic differences,^29^ public datasets of normal controls for early-phase imaging are desirable, largely encouraged and in development by the European Association of Nuclear Medicine (EANM). In this view, formal training of physicians in the field would also be necessary for perfusion PET scans.^27^ Moreover, it is important to consider that perfusion images as well as ^18^F-FDG-PET can suffer from biases in the presence of severe atrophy and cerebral vascular disease.^11,24^ Thus, structural images acquired in combination with PET or close to it are crucial for adequate image interpretation. In addition, blood glucose level limits ^18^F-FDG uptake in the brain and thus its use in diabetic patients,^30^ and its effect on early-phase amyloid scanning, which is expected to be lower, should be explored.

Lastly, the lack of reimbursement for amyloid PET can represent a limit for its wide clinical implementation for some countries that needs to be taken into account.

Overall, the possibility of acquiring information about amyloidosis and neurodegeneration with a single tracer injection offered by dual-phase PET protocol represents a clear advantage in terms of radiation exposure and potentially in terms of costs. Multiple studies at group and individual levels support the readiness of dual-phase amyloid PET to be included in routine clinical practice, and emerging evidence is also paving the way toward achieving similar results for the early-phase imaging of tau PET.^31–34^
